# Development of a Protective Device to Prevent Damage to an Endotracheal Tube Caused by the Eruption of Deciduous Teeth

**DOI:** 10.3390/reports9030245

**Published:** 2026-07-28

**Authors:** Jimei Zhao, Kaoru Shigeta, Risa Yamamoto, Sara Watanabe, Ayako Chida-Nagai, Hirokuni Yamazawa, Tatsuya Akitomo, Koichi Nakamura

**Affiliations:** 1 Department of Pediatric Dentistry, Division of Oral Health and Development, Hiroshima University Hospital, Hiroshima 734-8553, Japan; 2 Division of Pediatric and Special Care Dentistry, Department of Oral Functional Science, Faculty of Dental Medicine, Hokkaido University, Sapporo 060-8586, Japan; 3 Department of Dental Medical Laboratory, Hokkaido University Hospital, Sapporo 060-8648, Japan; 4 Department of pediatrics, Hokkaido University Hospital, Sapporo 060-8648, Japan; 5 Department of Pediatric Dentistry, Graduate School of Biomedical and Health Sciences, Hiroshima University, Hiroshima 734-8553, Japan

**Keywords:** mechanical ventilation, endotracheal tube, inflation line, protective equipment, pediatric dentistry

## Abstract

In patients undergoing endotracheal intubation and mechanical ventilation, damage to the endotracheal tube or inflation line is a potential risk with life-threatening consequences. Although various devices and bite blocks have been developed to secure the endotracheal tube and prevent its dislodgement or damage, they are not suitable for children or newborns. We describe a 1-year-and-2-month-old girl who required long-term endotracheal intubation and mechanical ventilation. The eruption of the lower deciduous incisors caused damage to the inflation line. A mouthguard was not appropriate because her teeth had erupted only slightly, and the retention was insufficient. After collaborating with a dental technician to fabricate a protective device, we were able to provide the optimal treatment while minimizing invasiveness to the patient. It is necessary to collaborate with other professionals as a team to select the optimal medical care tailored to each patient.

**Figure 1:**
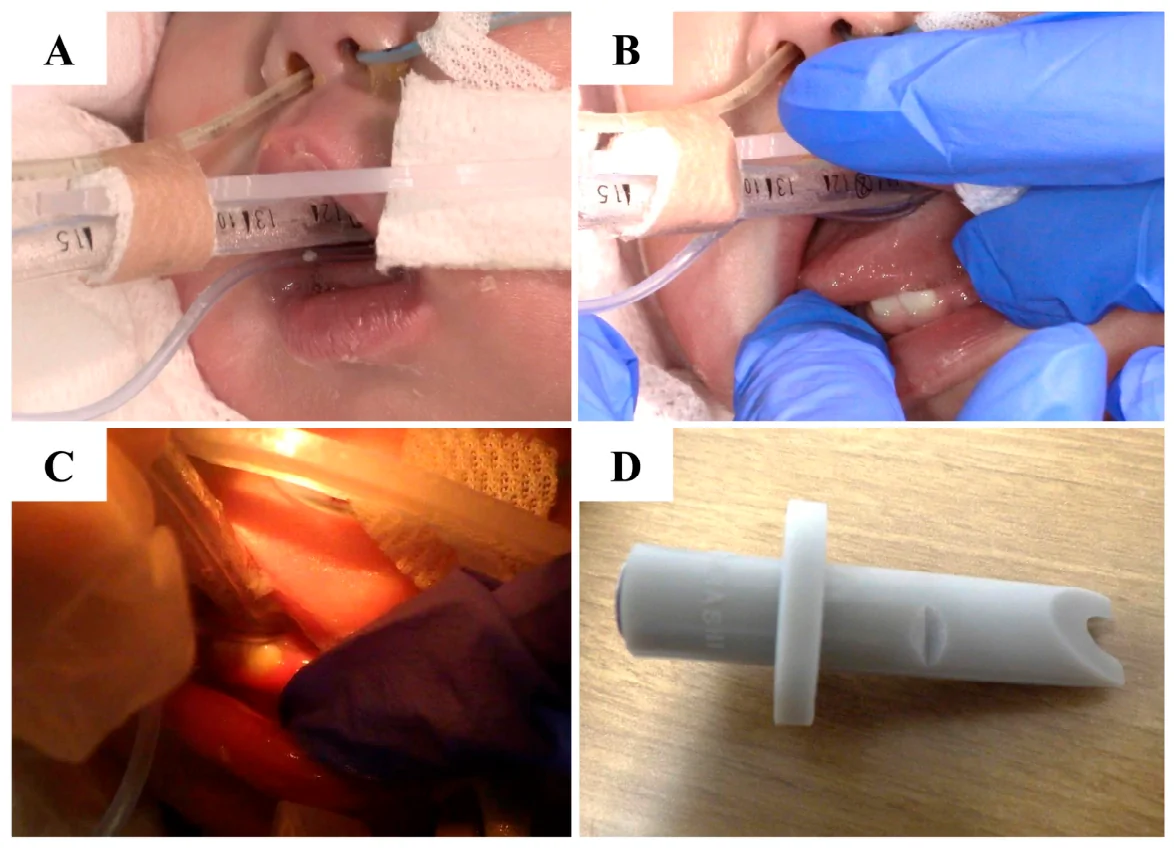
A 1-year-and-2-month-old girl was referred to our department to address endotracheal tube damage caused by her teeth (**A**). The patient had severe congenital heart disease (non-compaction of the ventricle, right ventricular hypoplasia, atrial septal defect, patent ductus arteriosus, tricuspid regurgitation, mitral regurgitation, and heart failure). She had required long-term mechanical ventilation owing to heart failure and had been on continuous intravenous sedation since birth. Tracheostomy had been deemed inappropriate because her overall condition had been critical. With the eruption of her lower central deciduous incisors bilaterally (**B**), the cutting edges began to contact the endotracheal tube, causing damage to the inflation line (**C**). For patients receiving mechanical ventilation, damage to the endotracheal tube or inflation line can pose serious airway-related risks, such as cuff leakage and ventilatory failure [1]. Off-the-shelf devices to secure the endotracheal tube were not available for a newborn. A bite block was used to prevent damage to the inflation line, and there were teeth marks on the bite block (**D**), suggesting a risk of pressure injury caused by the bite block. Although we attempted to create a mouthguard, the fit was poor because her teeth had erupted only slightly, and the retention was insufficient. Therefore, we consulted with dental technicians and decided to create and fit a protective device for the endotracheal tube itself, rather than using a mouthguard.

**Figure 2:**
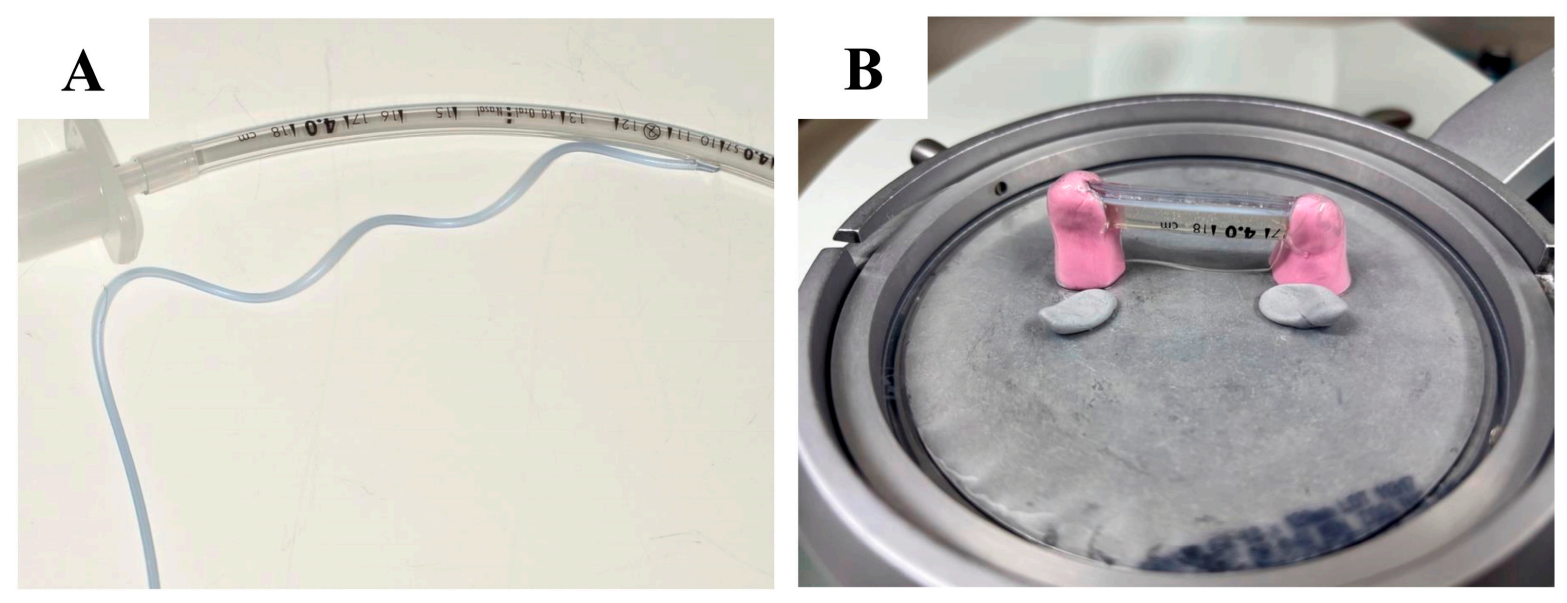
An endotracheal tube with 4 mm inner diameter was used (**A**). A protective device was fabricated and fitted directly onto the endotracheal tube and inflation line. Only the portions of the endotracheal tube and inflation line that were in contact with the teeth were cut out. Next, to prevent the tube from collapsing during the heat-sealing of the thermoplastic sheet in the subsequent step, the inner surface of the tube was reinforced with a self-curing resin (Unifast II; GC Corporation, Tokyo, Japan). The material was built up to a sufficient height so that the endotracheal tube and the inflation line formed a single unit and were completely covered (**B**). Next, a 120 × 1.0 mm thermoplastic sheet (Erkodent, Pfalzgrafenweiler, Germany), which is a material commonly used in the manufacture of mouthpieces, was heat-sealed over the top (heated for 50 s at a molding temperature of 160 degrees, then cooled under pressure for 45 s).

**Figure 3:**
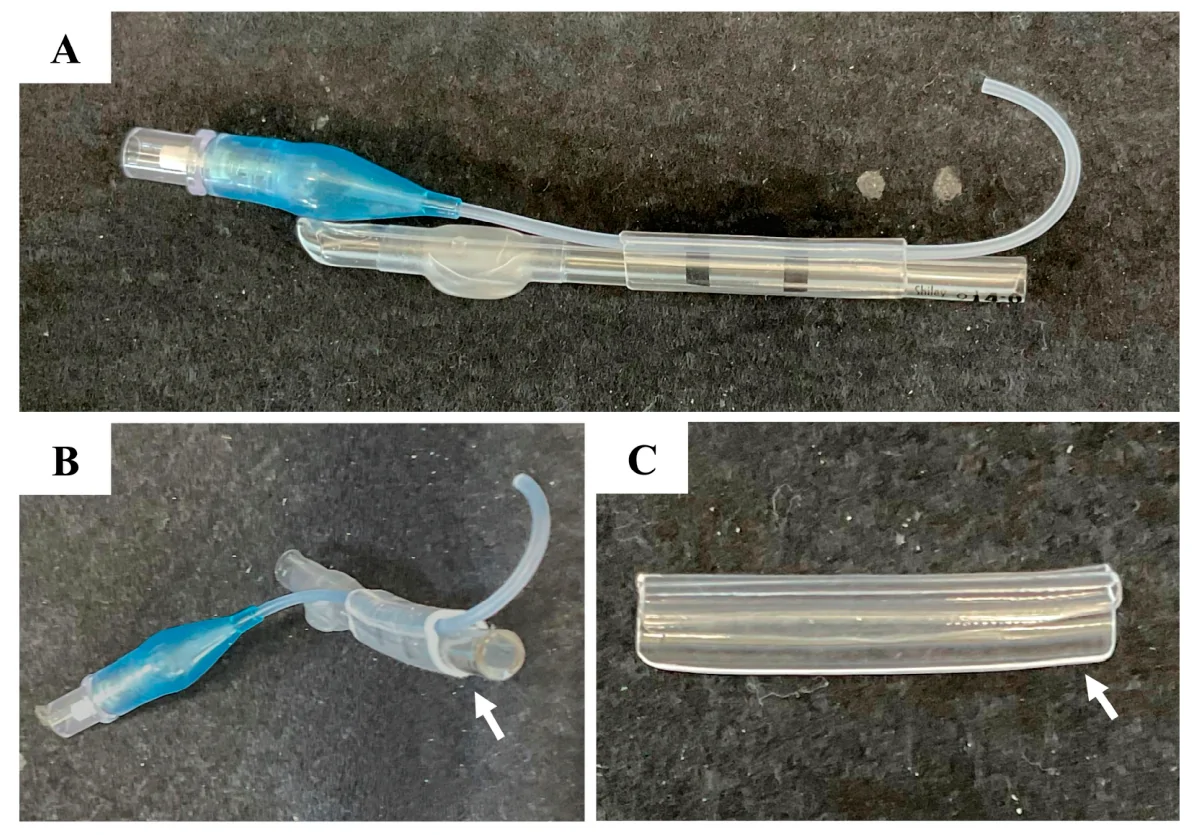
The endotracheal tube and inflation line were protected as a single unit (**A**). A cross-sectional view of the tube and inflation line is shown (**B**). A photo of the protective device on its own is shown (**C**). There is a slit at the bottom of the protective cover (opposite the inflation tube), allowing it to be attached and removed by opening the slit. The slit is indicated by arrows in (**B**,**C**).

**Figure 4:**
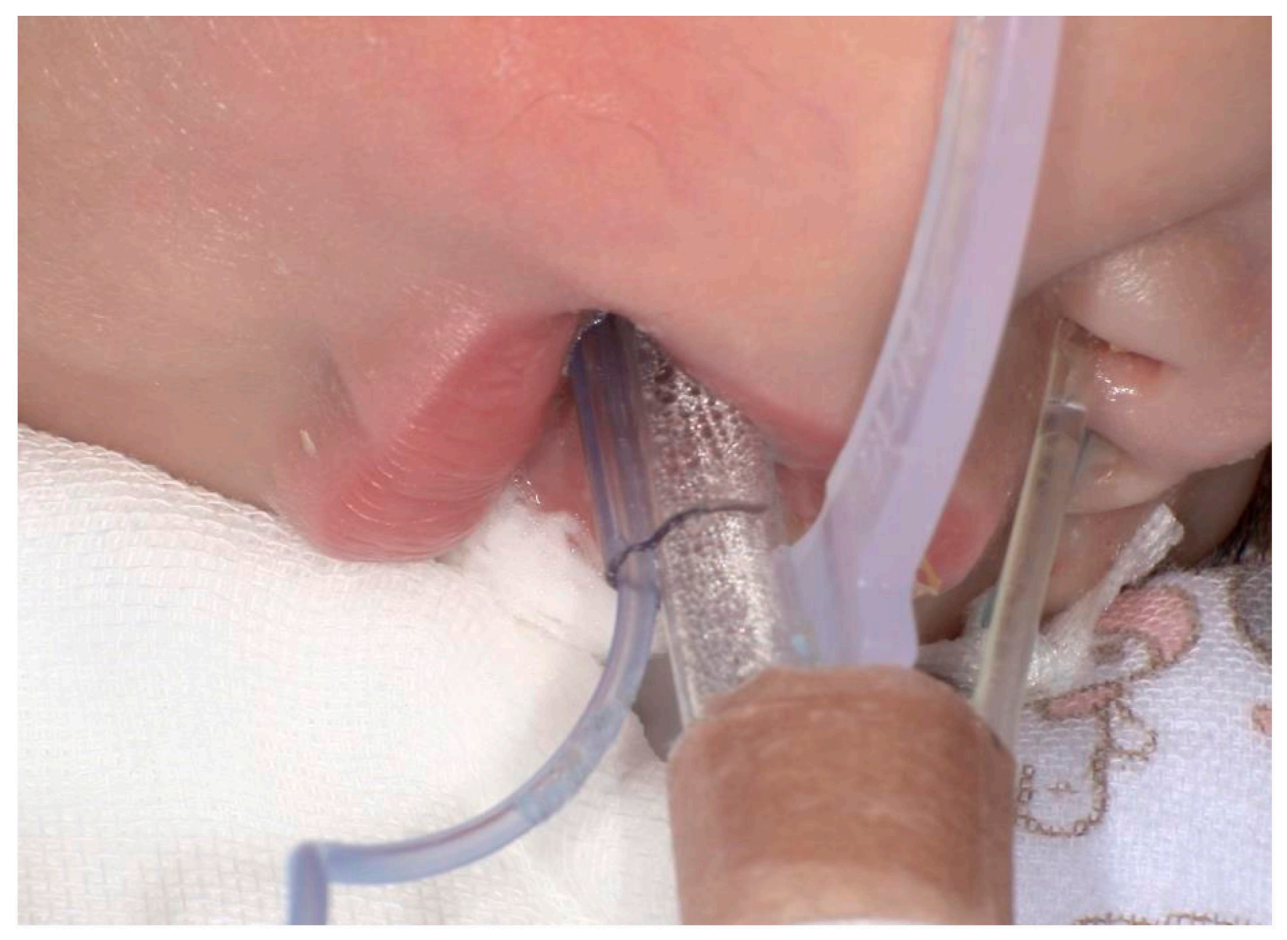
Nineteen days after the initial consultation, the protective device was fitted to the patient. The incisal edges of her central deciduous incisors were in contact with the device, and no compression, narrowing, or damage was observed in the endotracheal tube or the inflation line. The device was well-fitted to these instruments, and no spontaneous displacement or dislodgement was observed. The device was applied by a healthcare professional, who also performed all subsequent attachment and removal procedures. Six months after the initial consultation, a tracheostomy was performed when the patient was 1 year and 8 months old. The protective device was used continuously for 6 months, and no complications such as tube damage, oral mucositis, or pressure ulcers were observed. Over the past decade, as medical care in neonatal and pediatric intensive care units has improved, more children have been receiving mechanical ventilation [2]. Because endotracheal intubation carries a risk of spontaneous dislodgement, long-term intubation is not common; however, there have been reports of cases, such as this one, where long-term intubation was performed because a tracheostomy was not indicated [3,4]. For patients receiving mechanical ventilation via endotracheal intubation, damage to the intubation pathway can pose a risk. In this case, damage to the inflation line became apparent as the teeth erupted. Various devices, such as the AnchorFast Oral Endotracheal Tube Fastener (Hollister Incorporated, Libertyville, IL, USA) have been developed to secure the endotracheal tube and prevent its dislodgement or damage [5,6,7,8]. Although the range of such devices available for pediatric patients is limited, Yano et al. have reported on the use of AnchorFast in a 5-year-old patient [9]. However, such devices are not suitable for neonatal tubes with small inner diameters. There are also bite blocks such as Universal Bite Block (B&B Medical Technologies, Carlsbad, CA USA) available to protect endotracheal tubes in pediatric patients, but prolonged use may lead to pressure ulcers. Moreover, there was a report of the fabrication of a device to secure the endotracheal tube integrated with a mouthguard [10]. In our case, it was difficult to make a mouthguard because the teeth had erupted only slightly, and retention was insufficient. Although occlusal adjustment or extraction of the lower bilateral central deciduous incisors was considered as another treatment option to prevent damage to the endotracheal tube or inflation line, we wished to avoid aggressive intervention. Additionally, since the patient was only 14 months old and was expected to continue teething, it was necessary to consider how to manage newly erupting deciduous teeth in the future. By collaborating with a dental technician, we were able to provide optimal treatment while minimizing invasiveness to the patient. Subsequently, the upper bilateral central deciduous incisors also began to erupt, and the process proceeded without any complications. Although this is not a typical case, it is necessary to collaborate with other professionals as a team to select optimal medical care tailored to each patient. This report is limited to just a single patient, and it is uncertain whether this approach is generally applicable in clinical practice. However, it has the potential to be an option for similar cases in the future.

## Data Availability

The original contributions presented in this study are included in the article. Further inquiries can be directed to the corresponding author.
